# Bakuchiol protects against pathological cardiac hypertrophy by blocking NF-κB signaling pathway

**DOI:** 10.1042/BSR20181043

**Published:** 2018-10-31

**Authors:** Zheng Wang, Lu Gao, Lili Xiao, Lingyao Kong, Huiting Shi, Xinyu Tian, Luosha Zhao

**Affiliations:** Department of Cardiology, The First Affiliated Hospital of Zhengzhou University, Zhengzhou, China

**Keywords:** aortic banding, Bakuchiol, cardiac hypertrophy, cardiomyocytes, NF kappa B

## Abstract

Bakuchiol (Bak), a monoterpene phenol isolated from the seeds of Psoralea corylifolia, has been widely used to treat a large variety of diseases in both Indian and Chinese folkloric medicine. However, the effects of Bak on cardiac hypertrophy remain unclear. Therefore, the present study was designed to determine whether Bak could alleviate cardiac hypertrophy. Mice were subjected to aortic banding (AB) to induce cardiac hypertrophy model. Bak of 1 ml/100 g body weight was given by oral gavage once a day from 1 to 8 weeks after surgery. Our data demonstrated for the first time that Bak could attenuate pressure overload-induced cardiac hypertrophy and could attenuate fibrosis and the inflammatory response induced by AB. The results further revealed that the effect of Bak on cardiac hypertrophy was mediated by blocking the activation of the NF-κB signaling pathway. *In vitro* studies performed in neonatal rat cardiomyocytes further proved that the protective effect of Bak on cardiac hypertrophy is largely dependent on the NF-κB pathway. Based on our results, Bak shows profound potential for its application in the treatment of pathological cardiac hypertrophy, and we believe that Bak may be a promising therapeutic candidate to treat cardiac hypertrophy and heart failure.

## Introduction

Cardiac hypertrophy develops as an important compensatory mechanism of the heart to improve the conditions of hemodynamic overload caused by diverse pathological states, such as aortic stenosis, valvular diseases, myocardial infarction, and hypertension [1,2]. Cardiac hypertrophy is characterized by an increase in myocardial mass and protein synthesis, by the abnormal expression of fetal genes and by the excessive deposition of extracellular matrix [3]. Cardiac hypertrophy is initially a beneficial adaptive response that maintains normal cardiac function; however, sustained cardiac hypertrophy ultimately progresses to congestive heart failure [4]. Epidemiological studies have demonstrated that cardiac hypertrophy is an important independent risk factor for heart failure, malignant arrhythmia, and sudden death [5]. In recent decades, multiple signaling pathways have been studied in the hypertrophic process, such as the mitogen-activated protein kinase (MAPK) pathway, the calcineurin/nuclear factor of activated T-cells (NFAT) pathway, and the PI3K/Akt pathway [4,6,7]. Inhibition of these signaling pathways will eventually attenuate cardiac hypertrophy by regulating multiple transcription factors that alter gene expression. Therefore, pharmacological interventions of these signal transduction pathways may provide ideal approaches for treating cardiac hypertrophy. However, effective drugs targeting the signal transduction pathways involved in cardiac hypertrophy have not been found. The current challenge will be to find promising pharmacological agents that selectively modulate the specific signaling pathways and thus alleviate pathological cardiac hypertrophy.

Bakuchiol (1-(4-hydroxyphenyl)-3,7-dimethyl-3-vinyl-1,6-octa-diene) (Bak), a monoterpene phenol isolated from the seeds of Psoralea corylifolia, has been widely used to treat a large variety of diseases in both Indian and Chinese folkloric medicine [8,9]. In a previous study, Li et al. [10] reported that Bak exhibited a potent hazardous effect on liver lipid metabolism, and they found that the mechanisms involved might be attributed to the disordered lipid metabolism homeostasis of hydroxymethylglutaryl-CoA (HMG-CoA) by the RohA pathway and of peroxisome proliferator-activated receptor α (PPARα) by inducing the expression of liver X receptor α (LXRα). In addition, Cheng and co-workers found that Bak induced S-phase arrest in breast cancer cells through the inactivation of Cdc2 and that it induced cell apoptosis via the intrinsic mitochondrial pathway, which showed the potential of Bak as an anti-breast cancer drug, as well as its potential to be used in HRT for relieving menopausal symptoms [11]. In rat liver myofibroblasts, Bak induced caspase-3-dependent apoptosis through the activation of c-Jun N-terminal kinase (JNK). In addition, Bak has anti-inflammatory activity since it is capable of controlling leukocytic functions [12]. More recently, it has been demonstrated that Bak could attenuate myocardial ischemia reperfusion injury (IRI) by activating SIRT1/PGC-1α signaling, suggesting its potential protective role in the cardiovascular system [13]. However, the effects of Bak on cardiac hypertrophy remain unclear. Therefore, the present study was designed to determine whether Bak could alleviate pathological cardiac hypertrophy and to explore the underlying molecular mechanisms.

In the present study, we observed that Bak could attenuate pressure overload-induced cardiac hypertrophy and could attenuate fibrosis and the inflammatory response induced by aortic banding (AB). Mechanistically, we found that Bak plays a crucial role in the protective effects of cardiac hypertrophy by NF-κB signaling suppression. Based on our results, we believe that Bak may be a promising therapeutic candidate to treat cardiac hypertrophy and heart failure.

## Materials and methods

### Reagents

Bak (purity ≥98%) was purchased from the Shanghai Winherb Medical Co. (Shanghai, China). Antibodies against the following proteins were purchased from Cell Signaling Technology: inhibitor of κB kinase-β (IKKβ) (#2370), p-IκBα ^ser32/36^ (#9246), IκBα (#4814), p-p65^ser536^ (#3033), p65 (#4764), tumor necrosis factor-α (TNF-α) (#3707), monocyte chemoattractant protein 1 (MCP-1) (#2029), interleukin-6 (IL-6) (#12912), GAPDH (#2118). TRIzol reagent and the RT-PCR Kit were purchased from Life Technologies, Inc. (Gaithersburg, MD, U.S.A.). The BCA Protein Assay Kit and anti-α-actin were obtained from Pierce (Rockford, IL, U.S.A.). All the other chemicals were purchased from Sigma (St. Louis, MO, U.S.A.).

### Animals and AB operation

The male C57BL/6J mice used in the present study were obtained from the Institute of Laboratory Animal Science, Chinese Academy of Medical Sciences (Beijing, China). All of the animal procedures were approved by the Animal Care and Use Committees of the First Affiliated Hospital of Zhengzhou University. The mice were randomly assigned to four groups. The Bak suspension was prepared using a 0.5% carboxy methylcellulose solution and was administered at a constant volume of 1 ml/100 g body weight (BW) by oral gavage once a day from 1 to 8 weeks after surgery. The control group was given the same volume of the vehicle solution (0.5% carboxy methylcellulose).

AB was performed as described in detail previously [14]. In brief, after the mice were anesthetized by intraperitoneal injection of sodium pentobarbital (50 mg/kg, Sigma), the left chest was opened, and the descending thoracic aorta was identified. Then, the descending thoracic aorta was ligated with a 27G or 26G needle with a 7-0 silk suture. A similar sham operation was performed in the sham-operated mice but without aortic ligation.

### Neonatal rat cardiomyocytes culture

Neonatal rat cardiac myocytes (NRCMs) were prepared as described previously [15]. In brief, the cells were cultured in Dulbecco’s Modified Eagle Medium (DMEM), supplemented with 20% FBS, streptomycin (100 mg/ml) and penicillin (100 U/ml), and were grown in a humidified CO_2_ incubator with 5% CO_2_ at 37°C. The cells were seeded into six-well culture plates at a density of 1 × 10^6^ per well before being incubated for 24 h. Then, the cells were starved for 12 h. Ang II (1 μM) in the absence or presence of different concentrations of Bak (1, 5, and 10 μM) was added to the medium, and the cells were incubated for another 24 h.

### Western blotting

The proteins extracted from the different groups were determined using the BCA Protein Assay Kit. Then, the proteins were loaded into SDS–PAGE gels and transferred to a PVDF membrane (Millipore, #ISEQ00010). The membrane was blocked with 5% nonfat milk and was then incubated with different primary antibodies overnight at 4°C. After incubation with different primary antibodies, the membrane was incubated with secondary antibody at room temperature for 2 h. The blots were scanned using an Odyssey Infrared Imaging System (Odyssey, LI‐COR). The specific protein expression levels were normalized to GAPDH levels.

### Immunofluorescence staining

In brief, the NRCMs were fixed with 3.7% formaldehyde in PBS for 15 min, permeabilized with 0.1% Triton X-100 in PBS for 40 min, and blocked with 5% BSA for 1 h at room temperature. Then, the cells were stained with an anti-α-actinin antibody at a dilution of 1:100 and were incubated with Alexa Fluor 488 goat anti-mouse IgG (Invitrogen, U.S.A.). Finally, Slow Fade Gold antifade reagent with DAPI (Invitrogen, U.S.A.) was used for counter staining. The surface areas were measured using Image-Pro Plus 6.0 software.

### Echocardiography

MyLab 30CV Ultrasound (Biosound Esaote Inc.) was used for echocardiography as previously described [16]. After the mice were anesthetized with 1.5–2% isoflurane, the parameters of the mouse heart were measured from both the parasternal short-axis view and the parasternal long-axis view at a frame rate of 120Hz. The left ventricular (LV) end-systolic diameter (LVDs) and the LV end-diastolic dimension (LVDd) were measured from the LV M-mode tracing with a sweep speed of 50 mm/s at the midpapillary muscle level.Fractional shortening (FS) was calculated by the following formula: FS = (LVDd − LVDs)/LVDd × 100%.

### Histological analysis

The hearts were excised from the anesthetized mice and were fixed for more than 24 h in 10% formalin after being arrested in a 10% potassium chloride solution, then they were embedded in paraffin. Subsequently, the hearts were transversely sectioned at 5 µm. The sections of each sample were stained with either hematoxylin–eosin (HE) for histopathology or picrosirius red (PSR) for collagen deposition. The myocyte cross-sectional area (CSA) and the LV collagen volume were measured with the quantitative digital image analysis system (Image-Pro Plus 6.0). In each group, more than 100 LV cardiomyocyte CSAs and more than 25 fields were measured.

### Quantitative real-time PCR

For real-time PCR, the total RNA was collected from mice ventricular tissues or cultured NRCMs using TRIZol (Invitrogen) according to the manufacturer’s instructions. To generate cDNA, the total RNA (2 μg) was reverse transcribed into cDNA using the Transcriptor First Strand cDNA Synthesis Kit (Roche). The mRNA levels of the selected genes were quantitated by real-time PCR using SYBR Green (Roche). The primers used are listed in Table 1, and the relative mRNA expression of the indicated genes was normalized to the GAPDH gene expression.

**Table 1:** Primers for real-time PCR

Gene name	Primers	Sequence
Rat ANP	Forward 5′-3′	AAAGCAAACTGAGGGCTCTGCTCG
	Forward 5′-3′	TTCGGTACCGGAAGCTGTTGCA
Rat BNP	Forward 5′-3′	GATAGACCGGATTGGCGCA
	Forward 5′-3′	GTGGCAAGTTTGTGCTGGAAGA
Rat β-MHC	Forward 5′-3′	TCTGGACAGCTCCCCATTCT
	Forward 5′-3′	CAAGGCTAACCTGGAGAAGATG
Mouse ANP	Forward 5′-3′	ACCACCTGGAGGAGAAGA
	Forward 5′-3′	TTCAAGAGGGCAGATCTATC
Mouse BNP	Forward 5′-3′	TCCAGCAGAGACCTCAAAATTCC
	Forward 5′-3′	TCAAAGGTGGTCCCAGAGCT
Mouse β-MHC	Forward 5′-3′	CCGAGTCCCAGGTCAACAA
	Forward 5′-3′	CTTCACGGGCACCCTTGGA
Mouse Collagen-α	Forward 5′-3′	TGGTACATCAGCCCGAAC
	Forward 5′-3′	GTCAGCTGGATAGCGACA
Mouse Collagen-α	Forward 5′-3′	CCCAACCCAGAGATCCCATT
	Forward 5′-3′	GAAGCACAGGAGCAGGTGTAGA
Mouse CTGF	Forward 5′-3′	TGACCCCTGCGACCCACA
	Forward 5′-3′	TACACCGACCCACCGAAGACACAG
Mouse MCP-1	Forward 5′-3′	GCTACTCATTCACCAGCAAGAT
	Forward 5′-3′	GGTGCTGAAGACCTTAGGGC
Mouse TNF-α	Forward 5′-3′	CGAGTGACAAGCCTGTAGCC
	Forward 5′-3′	GGCAGCCTTGTCCCTTGAAG
Mouse IL-6	Forward 5′-3′	CCTGGAGTACATGAAGAACAAC
	Forward 5′-3′	AGTGAGGAATGTCCACAAACT
Rat GAPDH	Forward 5′-3′	ATGATGACATCAAGAAGGTGGTG
	Forward 5′-3′	ATGTAGGCCATGAGGTCCAC
Mouse GAPDH	Forward 5′-3′	ACTCCACTCACGGCAAATTC
	Forward 5′-3′	TCTCCATGGTGGTGAAGACA

Abbreviations: β-MHC, β-myosin heavy chain; ANP, atrial natriuretic peptide; BNP, B-type natriuretic peptide; CTGF, connective tissue growth factor.

### Statistical analysis

Data were expressed as the means ± S.E.M. Differences among the groups were compared by two-way analysis of variance, followed by the *post hoc* Tukey test. Comparisons between two groups were assessed by an unpaired Student’s *t* test. *P*<0.05 was considered statistically significant. Statistical analyses were performed using SPSS software (version 17.0, SPSS Inc.).

### Ethics approval and consent to participate

The experimental protocols were approved by the Animal care and Use Committee of The First Affiliated Hospital of Zhengzhou University.

## Results

### Bak protects against angiotensin II-induced cardiomyocyte hypertrophy *in vitro*

**Figure 1:**
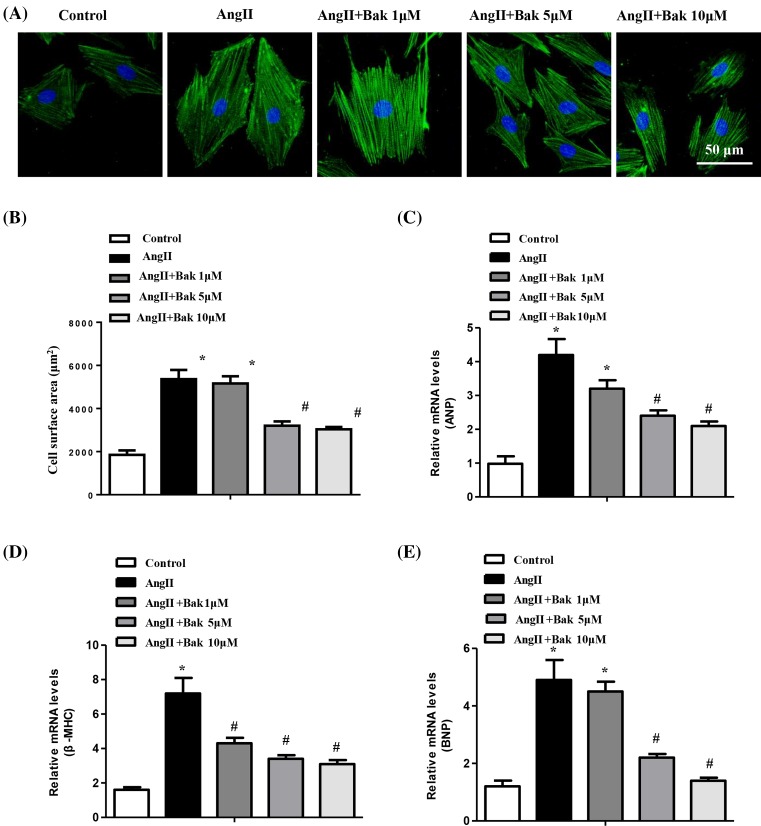
Bak inhibits cardiomyocyte hypertrophy *in vitro* (**A**) Representative images of NRCMs pretreated with different concentrations of Bak for 48 h and subsequently treated with 1 μM Ang II for 24 h (*n* = 3 independent experiments; blue, nucleus; green, α-actinin; scale bar, 50 μm). (**B**) Individual cell surface area of at least 100 NRCMs per group were traced and compared between the indicated groups (*P<0.05 versus control; ^#^*P*<0.05 versus Ang II-treated cells). (**C**–**E**) The relative mRNA levels of the hypertrophic markers ANP, BNP, and β-MHC were analyzed by real-time PCR and compared between the indicated groups (*P<0.05 versus control; ^#^*P*<0.05 versus Ang II-treated cells).

To determine the effects of Bak on cardiac hypertrophy *in vitro*, we used Ang II to induce cardiomyocyte hypertrophy. Cultured NRCMs were pretreated with Bak at the indicated concentrations for 24 h and were subsequently stimulated with Ang II for 48 h, followed by immunostaining with α-actinin to measure the cell size; mRNA levels were evaluated for atrial natriuretic peptide (ANP), B-type natriuretic peptide (BNP), and β-myosin heavy chain (β-MHC) markers of the hypertrophic hearts. The results showed that Bak treatment significantly inhibited the enlargement of the cardiomyocytes induced by Ang II *in vitro*, especially at a concentration of 10 μM (Figure 1A,B). In addition, further experiments revealed that Bak markedly reduced the induction of ANP, BNP, and β-MHC mRNA expression by Ang II (Figure 1C–E). Therefore, these data indicated that AngII-induced cardiac hypertrophy could be effectively suppressed by Bak, especially at a concentration of 10 μM.

### Bak attenuates pressure overload-induced cardiac hypertrophy and improves impaired cardiac function

**Figure 2:**
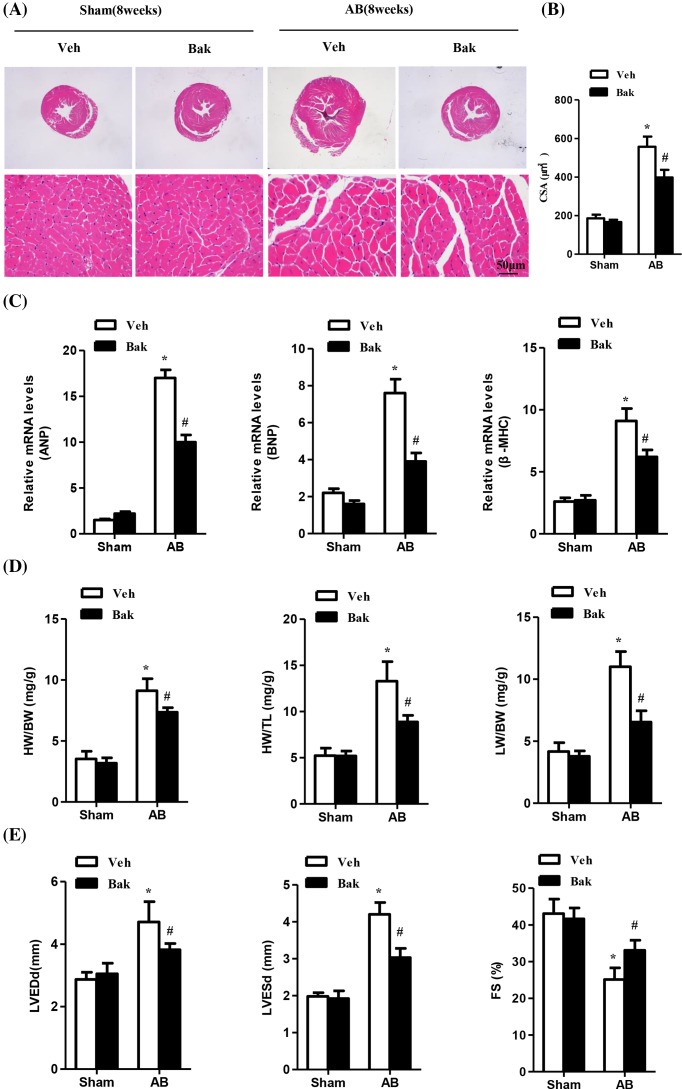
Bak attenuates pressure overload-induced cardiac hypertrophy and improves impaired cardiac function (**A**) Representative images of sections subjected to HE staining from vehicle (Veh) or Bak-treated mice 8 weeks after sham or AB surgery (*n* = 5–6 mice per experimental group; scale bar, 50 μm). (**B**) The statistical results of cardiomyocyte CSAs in each group (*n*≥100 cells; *P<0.05 versus vehicle/sham, ^#^*P*<0.05 versus vehicle/AB after AB). (**C**) Real-time PCR analyses of the hypertrophy markers ANP, BNP, and β-MHC induced by AB or sham surgery in each group of mice (*n*=5; *P<0.05 versus vehicle/sham; ^#^*P*<0.05 versus vehicle/AB after AB). (**D**) The statistical results of the HW/BW, HW/TL, and LW/BW ratios in the indicated groups (*n*=5; *P<0.05 versus vehicle/sham; ^#^*P*<0.05 versus vehicle/AB after AB). (**E**) Statistical results of the echocardiographic parameters of the four groups of mice after 8 weeks of AB or sham operations (*n*=5; *P<0.05 versus vehicle/sham; ^ #^*P*<0.05 versus vehicle/AB after AB).

Based on our former results that Bak effectively suppressed cardiomyocyte hypertrophy induced by Ang II, we next sought to determine whether Bak could antagonize AB-induced cardiac hypertrophy. The mice were randomly assigned to four groups. The Bak-treated mice and vehicle-treated mice were subjected to either AB or the sham operation. Of note, the mice treated with isorhamnetin showed no apparent phenotypic character alterations in cardiac function or structure compared with vehicle-treated mice under the basal condition. After 8 weeks of AB, the AB-induced vehicle-treated mice developed massive cardiac hypertrophy as shown by an increased LV wall and cardiomyocyte CSA accessed by HE; up-regulation of the hypertrophic markers ANP, BNP, and β-MHC; and heart weight (HW)/BW, lung weight (LW)/BW, and HW/tibia length (TL) ratios. However, compared with the AB-induced vehicle-treated mice, the myocardial hypertrophic response was significantly blocked in isorhamnetin-treated mice (Figure 2 and Table 2).

**Table 2:** Parameters in Veh- and Bak-treated mice at 8 weeks after sham or AB operation

Parameters	Veh/sham	Bak/sham	Veh/AB	Bak/AB
BW, g	27.65 ± 1.86	28.33 ± 2.87	26.99 ± 3.02	27.01 ± 1.98
HW/BW, mg/g	3.53 ± 0.62	3.18 ± 0.44	9.11 ± 0.99*	7.35 ± 0.38^*†^
LW/BW, mg/g	4.17 ± 0.71	3.78 ± 0.43	11.0 ± 1.22*	6.54 ± 0.91^*^†^^
HW/TL, mg/mm	5.22 ± 0.82	5.19 ± 0.53	13.28 ± 2.11*	8.87 ± 0.72^*^†^^
HR, bpm	534 ± 34	529 ± 43	504 ± 55	501 ± 48
IVSd, mm	0.69 ± 0.06	0.71 ± 0.10	0.93 ± 0.08*	0.82 ± 0.06^†^
LVDd, mm	2.87 ± 0.23	3.05 ± 0.34	4.71 ± 0.65*	3.82 ± 0.20^*^†^^
LVPWd, mm	0.72 ± 0.04	0.68 ± 0.03	0.93 ± 0.07*	0.86 ± 0.08
IVSs, mm	0.92 ± 0.08	1.01 ± 0.07	1.21 ± 0.13*	1.12 ± 0.09
LVDs, mm	1.98 ± 0.10	1.92 ± 0.21	4.2 ± 0.32*	3.03 ± 0.25^*^†^^
LVPWs, mm	1.05 ± 0.11	0.97 ± 0.07	1.32 ± 0.12*	1.13 ± 0.05^†^
EF, %	79.23 ± 3.25	77.98 ± 4.27	53.22 ± 2.88*	67.14 ± 3.0^*^†^^
FS, %	43.07 ± 3.94	41.65 ± 2.96	25.13 ± 3.17*	33.09 ± 2.75^*^†^^

All values are presented as mean ± S.E.M. EF, ejection fraction; IVSd, end-diastolic interventricular septum thickness; IVSs, end-systolic interventricular septum thickness; LVPWd, end-diastolic LV posterior wall thickness; LVPWs, end-systolic LV posterior wall thickness; Veh, vehicle. (*P<0.05 versus vehicle/sham; ^#^*P*<0.05 versus vehicle/AB after AB).

Consistent with these findings, the vehicle-treated mice exhibited cardiac dilation and dysfunction 8 weeks after AB, as measured by echocardiographic parameters including LV end-diastolic dimension (LVDd), LV end-systolic dimension (LVDs), and LVFS. However, this pathological cardiac growth, which was induced by chronic pressure overload, was remarkably attenuated in the Bak-treated group after 8 weeks of AB surgery (Figure 2E and Table 2). Taken together, these data implied that Bak could attenuate pressure overload-induced cardiac hypertrophy and could improve impaired cardiac function.

### Bak attenuates fibrosis and the inflammatory response induced by AB

**Figure 3:**
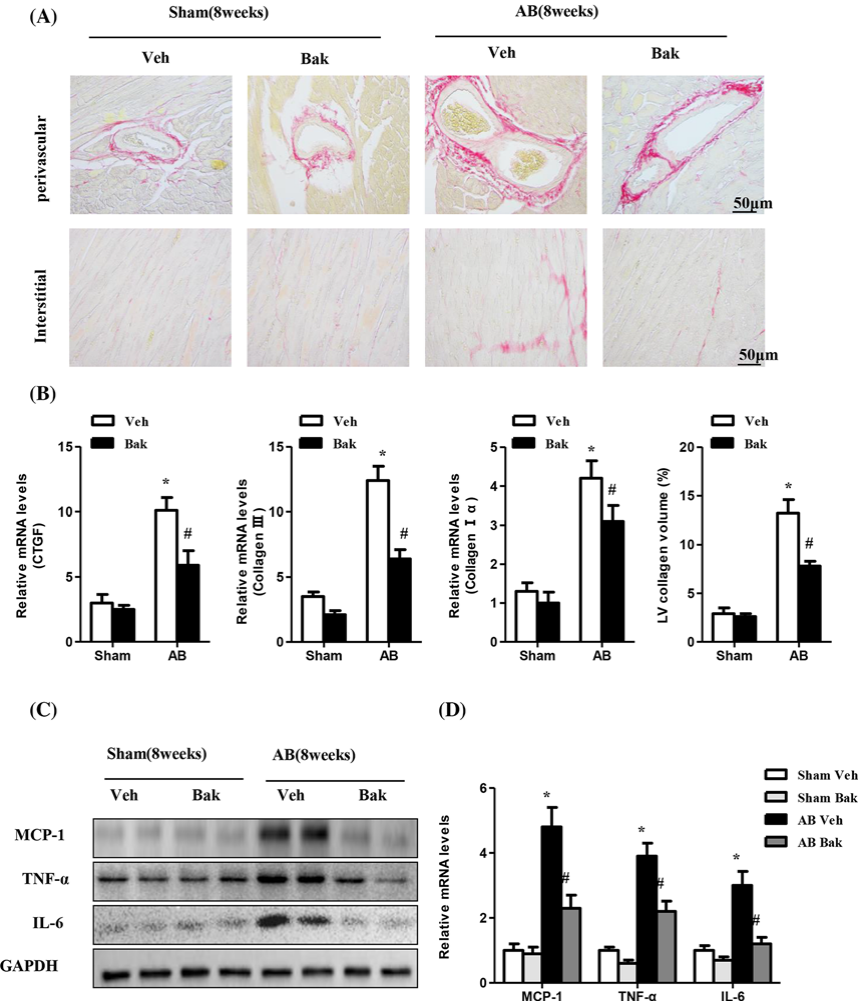
Bak attenuates fibrosis and inflammatory responses induced by AB (**A**) Representative images of sections subjected to PSR staining from Veh- or Bak-treated mice 8 weeks after sham or AB surgery (*n* = 5–6 mice per experimental group; scale bar, 50 μm). (**B**) The statistical results of LV collagen volume in each group (*n*≥25 fields from 5 mice per experimental group; *P<0.05 versus vehicle/sham; ^#^*P*<0.05 versus vehicle/AB after AB). (**C**) Representative Western blots and quantitative results (**D**) showing MCP-1, TNFα, and IL-6 expression in heart tissues of mice in the indicated groups (*n*=5; *P<0.05 versus vehicle/sham; ^#^*P*<0.05 versus vehicle/AB after AB).

Pathological cardiac hypertrophy is accompanied by increased fibrosis and is characterized by the accumulation of collagen in the heart. To further define the effect of Bak on maladaptive cardiac remodeling, we examined the effect of Bak on cardiac fibrosis. The extent of fibrosis was assessed by quantitating the collagen volume in the interstitial and perivascular spaces. As expected, fibrosis in the interstitial and perivascular spaces was dramatically increased in vehicle-treated hearts that were subjected to AB but was markedly attenuated in Bak-treated hearts that experienced the same treatment (Figure 3A). We also measured the synthesis of collagen by analyzing the mRNA levels of fibrotic markers including collagen Iα, collagen III, and connective tissue growth factor. Consistent with the collagen volume, mRNA levels of fibrotic markers also decreased in Bak-treated hearts compared with the vehicle-treated mice induced by chronic pressure overload (Figure 3B). Taken together, these data imply that Bak suppressed pressure overload–induced cardiac remodeling.

In a previous study, Zhang et al. [17] demonstrated the *in vitro* and *in vivo* inhibitory effects of Bak on inflammation. To determine whether Bak prevents inflammatory responses in the heart, cytokine induction was characterized by Western blotting and q-PCR analyses. As expected, Bak-treated mice had significantly decreased TNF-α, IL-6, and MCP-1 levels, as well as mRNA levels, in cardiac tissue after 8 weeks of surgery compared with the vehicle-treated mice (Figure 3C,D).

Apoptosis is a mechanism by which cells can be eliminated without an inflammatory response. Evidence of an increased rate of apoptosis has been detected in hearts with experimentally induced hypertrophy and cardiomyopathy. We next examined the effects of Bak on apoptosis by TUNEL assays after 8 weeks of AB. Apoptotic cells were detected in Bak-treated mice and control mice; however, the fraction of apoptotic versus total cells in Bak-treated mice versus control mice showed no significant statistical difference after AB operation (data not shown).

### Bak mediates cardiac hypertrophy through the inhibition of the NF-κB pathway

**Figure 4:**
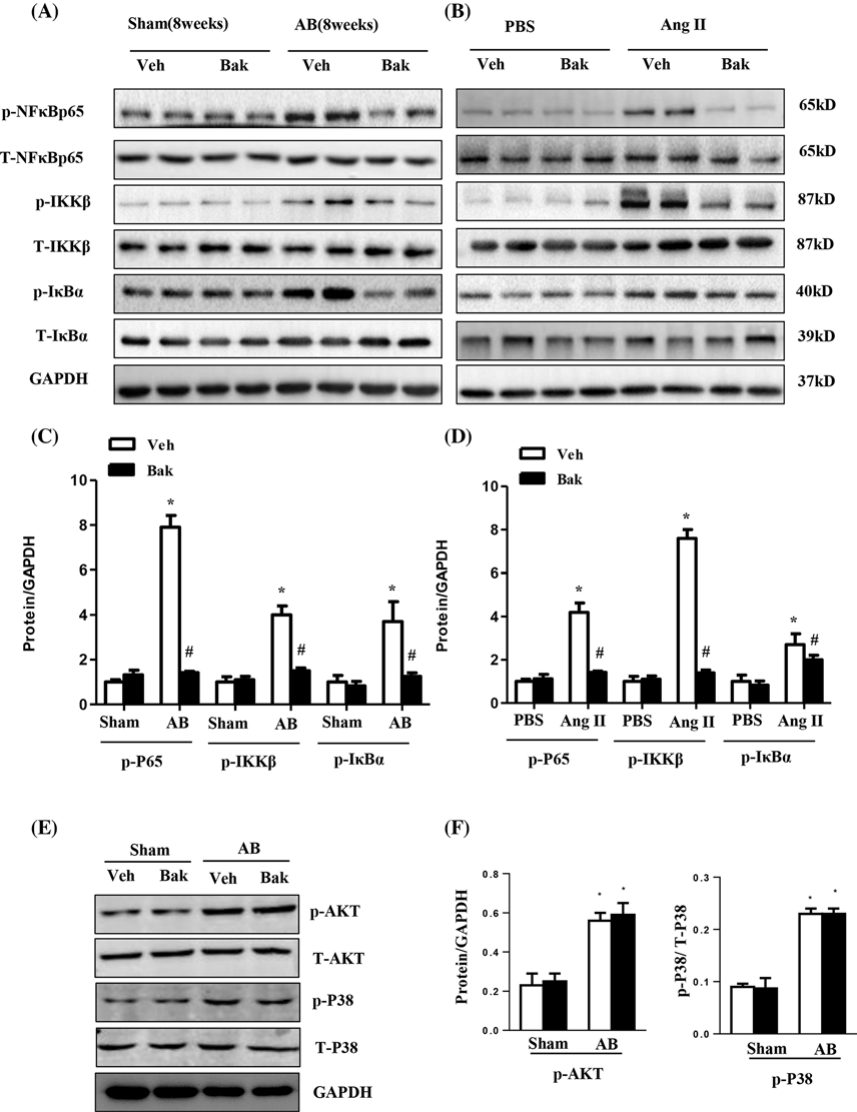
Bak mediates cardiac hypertrophy through the inhibition of the NF-κB pathway (**A**) Representative Western blotting analysis and quantitative results (**C**) showing phosphorylated and total NF-κB p65, IKKβ, and inhibitor of NF-κB a (IκBa) expression in heart tissues of mice in the indicated groups (*n*=5; *P<0.05 versus vehicle/sham; ^#^*P*<0.05 versus vehicle/AB after AB). (**B**) Representative Western blotting analysis and quantitative results (**D**) showing phosphorylated and total NF-κB p65, IKKβ and IκBa expression in NRCMs treated with Bak (10 μM) for 48 h subsequently treated with 1 μM Ang II for 24 h. (*P<0.05 versus control; ^#^*P*<0.05 versus AngII-treated cells) (**E** and **F**). Representative Western blotting analysis (E) and quantitative results (F) showing phosphorylated and total Akt and P38 expression in heart tissues of mice in the indicated groups (*n*=5; *P<0.05 versus vehicle/sham; ^#^*P*<0.05 versus vehicle/AB after AB).

To gain insight into the molecular mechanisms underlying the negative effects of Bak on pathological cardiac hypertrophy, we next sought to examine whether isorhamnetin affected the AB-induced activation of NF-κB signaling pathways. NF-κB activation, IKKβ and IκBα phosphorylation, as well as IκBα degradation, were clearly detected after 8 weeks of AB. As expected, NF-κB activation and phosphorylation were evidently blocked by Bak compared with vehicle-treated hearts after AB. Interestingly, Bak not only attenuated the phosphorylation and activation of NF-κB p65 but also inhibited the activation of IKKβ and IκBα (Figure 4A,C). Although the MAPK and PI3K/AKT signaling pathways play an important role in the conformation and development of cardiac hypertrophy, there was not much difference in the assessment of the activation of these pathways among the groups (data not shown). Subsequently, *in vitro* experiments were also performed. NRCMs were exposed to 1 μM of Ang II for 48 h. Our results showed that compared with the controls, Ang II-induced NF-κB activation was significantly reduced in the Bak-treated NRCMs (Figure 4B,D). These data indicate that Bak may exert its anti-hypertrophic effects through the inhibition of NF-κB signaling.

We also detected tha Akt and MAPK signaling pathway. As shown in Figure 4E,F, the Akt and P38 was activated after 8 weeks of AB, while Bak did not affect these signaling molecules.

### The protective effect of Bak on cardiac hypertrophy is largely dependent on the NF-κB pathway

**Figure 5:**
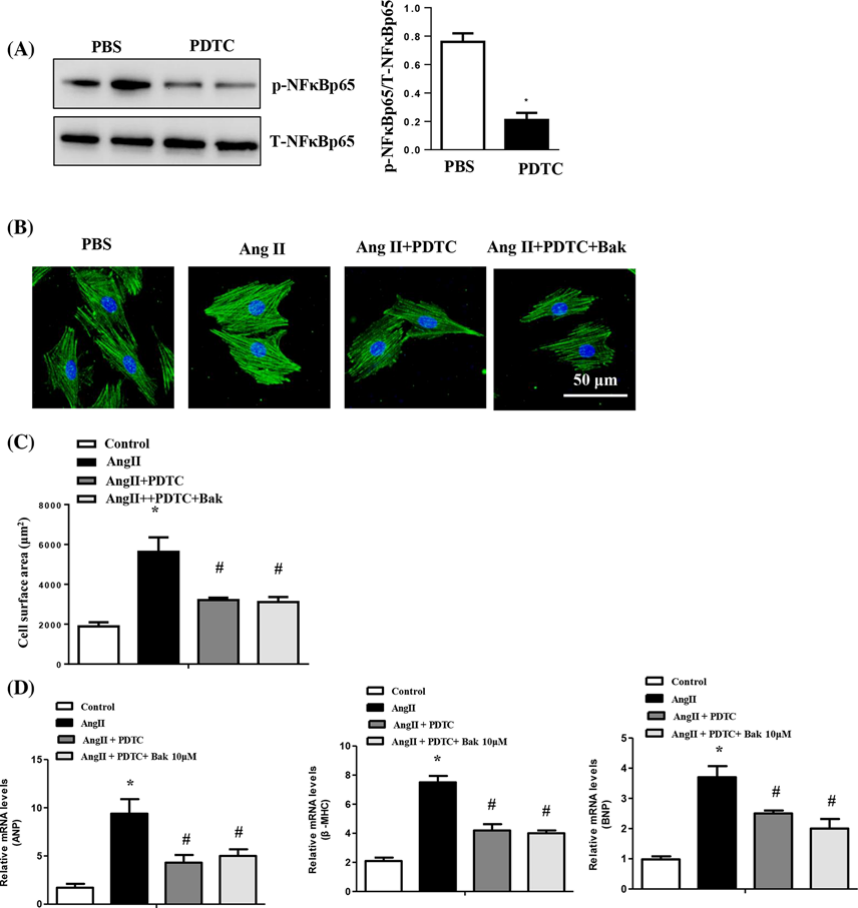
The protective effect of Bak on cardiac hypertrophy is largely dependent on the NF-κB pathway (**A**) Representative Western blotting analysis and quantitative results showing phosphorylated NF-κB p65 (*P<0.05 versus control). (**B**) Representative images of NRCMs that were pretreated with PDTC for 1 h and those that were subsequently treated with 1 μM Ang II for 48 h after being treated with Bak for 24 h. (**C**) Quantitation of the cell surface area (*n*=100 cells). (**D**) Real-time PCR analysis of the hypertrophy markers ANP, BNP, and β-MHC (*P<0.05 versus control; ^#^*P*<0.05 versus Ang II-treated cells).

To further confirm that Bak attenuates cardiac hypertrophy by mediating the NF-κB signaling pathway, we pretreated NRCMs with a selective NF-κB inhibitor, PDTC, for 1 h and then with Ang II for 48 h, after treatment with Bak for 24 h. As shown in Figure 5A, after PDTC treatment, the activation of NF-κB was decreased sharply. The NRCMs treated with Ang II showed pronounced hypertrophy, as assessed by cell surface area and by the expression levels of hypertrophic hallmarks (Figure 5B–D). The hypertrophic response was strongly blocked in the PDTC-treated cells compared with the cells treated with Ang II alone. However, PDTC (10 μM) did not affect the decreased hypertrophic response in the Bak-treated cells (Figure 5B–D). Collectively, these data suggest that the inhibition of cardiac hypertrophy by Bak is largely dependent on the inhibition of the NF-κB signaling pathway.

## Discussion

Pathological cardiac hypertrophy develops as an adaptive process in response to diverse pathological stimuli, associated with heart failure and malignant arrhythmia [1]. In the present study, our results suggest for the first time that Bak reverses the adverse effects of AB-induced pathological cardiac remodeling. Moreover, our observations demonstrated that Bak alleviated pathological cardiac hypertrophy through the inhibition of NF-κB signaling in response to pressure overload. We propose that Bak exerts cardioprotective effects in pathological cardiac hypertrophy in part through NF-κB signaling. Thus, Bak may be a promising therapeutic candidate for the intervention and prevention of heart failure.

Pathological cardiac hypertrophy is an important predecessor of heart failure, which is mainly characterized by cell enlargement, by the reactivation of fatal gene expression, by cardiac dysfunction in the extracellular matrix, and by hyperplasia of fibrosis. Although cardiac hypertrophy has long been considered an adaptive response to compensate for increased workload, prolonged hypertrophy is associated with an increased risk for malignant arrhythmia and heart failure, leading to increased cardiovascular mortality [1–4]. However, until now, there have been no effective drugs found to target the molecular changes involved in cardiac hypertrophy. Bak is a meroterpene found in the traditional Chinese herbal medicine Fructus psoraleae [8]. Kim et al. [18] found that Bak exhibits potent anti-cancer activity by directly targeting Blk, Hck, and p38 MAPK. Shoji et al. [19] reported that Bak produced enantiomer-selective anti-influenza A virus activity via a novel mechanism involving the host-cell response, which contributes to the development of novel approaches for the treatment of influenza. In addition, Seo et al. [20] demonstrated that Bak inhibits oxidative stress-induced mitochondrial dysfunction and ROS production. Furthermore, Choi et al. [21] observed that Bak is an effective anti-inflammatory compound, and Krenisky et al. [22] suggested a protective effect of Bak in the context of diabetes mellitus. Moreover, Feng et al. [13] reported that Bak treatment alleviates IRI by activating the SIRT1/PGC-1α signaling pathway, which indicates that Bak may play a protective role in the cardiovascular system. However, the effect of Bak on cardiac hypertrophy has not been clarified thus far. The results from our *in vitro* study showed that pretreatment of NRCMs with Bak protects NRCMs from Ang II-induced cardiomyocyte hypertrophy, which was verified by the decrease in cell surface area and the mRNA expression of ANP, BNP, and β-MHC. To further verify whether Bak could play a positive role in the progression of pathological cardiac remodeling, a surgical model of chronic pressure overload-induced pathological cardiac hypertrophy was established. As expected, the myocardial hypertrophic response was blocked in the Bak-treated mice. Importantly, a significant amelioration with improved cardiac function was observed in the Bak-treated mice in response to chronic pressure overload. It is well known that pathological cardiac hypertrophy is accompanied by increased fibrosis, which is characterized by the deposition of the extracellular matrix [23]. To further investigate whether Bak could ameliorate cardiac fibrosis, we detected the LV collagen volume and the mRNA expression levels of known mediators of fibrosis markers. Surprisingly, Bak attenuated the development of fibrosis in pressure overload-induced hearts. To further investigate the mechanism by which Bak ameliorates cardiac hypertrophy, we examined the effect of Bak on the cardiac inflammatory response induced by pressure overload stimuli. Our data showed that Bak-treated mice have significantly decreased inflammatory responses induced by AB. Thus, the administration of Bak may be a novel approach for the intervention and prevention of heart failure.

Recent evidence has shown that a number of intracellular signaling pathways including MAPK, NFAT, and PI3K/Akt, play pivotal roles in the development of cardiac hypertrophy [6]. These signaling pathways directly or indirectly induce hypertrophic growth via alteration of gene expression in the nucleus through activating a defined set of prohypertrophic transcription factors [6]. The underlying mechanisms by which Bak regulates cardiac remodeling remain largely unknown. Hence, the elucidation of the mechanisms underlying these characteristic phenomena related to Bak will potentially further our understanding of how Bak mediates cardiac hypertrophy. Given that the MAPK, PI3K/Akt, and NF-κB pathways have been proven to play an important role in the development of cardiac hypertrophy, we first examined whether Bak affected the AB-induced activation of the MAPK signaling pathway. However, there was not much difference in the assessment of MAPK activation among the groups. We then examined the PI3K/Akt signaling pathway, for which a role in cardiac hypertrophy is well established. However, Bak did not affect the phosphorylation and activation of Akt or its downstream targets mTOR and P70S6K. Considering that our data showed that Bak could inhibit cardiomyocyte hypertrophy, fibrosis, and inflammatory responses, the NF-κB signaling pathway was then tested. As expected, the cardiac hypertrophy induced by the AB operation was followed by the activation of the NF-κB signaling pathway (increased phosphorylation of NF-κB p65, IKKβ, and IκBa), and this activation was significantly down-regulated in the Bak-treated mouse hearts. Consistent with the results *in vivo*, Ang II-induced an increase in the phosphorylation of p65 (p-NF-κB p65), IKKβ (p-IKKβ) and IBa (p-IκBα), which were down-regulated in the Bak-pretreated NRCMs as well. To further ascertain whether Bak inhibits cardiac hypertrophy by inhibiting the NF-κB pathway, the NRCMs were treated with PDTC prior to Ang II and Bak treatments. However, PDTC treatment did not attenuate the hypertrophic response in the Bak-treated cells. Therefore, NF-κB signaling may be a critical pathway through which Bak mediates cardiac hypertrophy. All these findings demonstrated that Bak, with its ability to regulate NF-κB signaling, functioned as a protective and anti-hypertrophic mediator, regulating the pathogenesis of cardiac remodeling.

It is known that NF-κB signaling is indispensable for the regulation of a variety of physiological and pathophysiological processes, such as inflammation, fibrosis, cardiomyocyte hypertrophy, and ventricular remodeling. Some studies have revealed that NF-κB activation is required for the progression of cardiac hypertrophy. Recently, Hong et al. [24] reported that NF-κB inhibition with PDTC or p65 knockdown significantly decreased the hypertrophic responses. Moreover, IKK/NF-κB activation in cardiomyocytes caused significant inflammatory cardiomyopathy and heart failure, while inhibition of the NF-κB signaling pathway by a nondegradable IκBα decreased cardiac hypertrophy and dysfunction induced by Ang II stimuli and pressure overload [25]. However, there are some reports that have disputed the importance of the role of NF-κB signaling in the regulation of cardiac hypertrophy. For instance, Hikoso et al. [26] demonstrated that the cardio-specific knockdown of IKKβ promoted cardiac hypertrophy and heart failure in response to pressure overload. NF-κB inhibition by the cardiac-specific deletion of NEMO, a regulatory subunit of the IKK complex, augmented cardiac hypertrophy and cardiac dysfunction after pressure overload stimulation [27]. In the present study, we observed that the activation of NF-κB was inhibited in the heart by Bak, suggesting that Bak may attenuate cardiac remodeling through the negative regulation of NF-κB signaling. More importantly, we observed that the hypertrophic response was strongly blocked in the PDTC-treated cells compared with the cells treated with Ang II alone. However, PDTC did not affect the decreased hypertrophic response in the Bak-treated cells. Therefore, the inhibitory effects of Bak on cardiac hypertrophy may be largely dependent on the inactivation of NF-κB signaling. Given that NF-κB is likely to have multiple divergent effects and that it may promote or suppress pathogenic processes, future investigations are needed to determine whether Bak has therapeutic potential for the treatment of pathological cardiac hypertrophy. Given that cardiac hypertrophy is a complicated pathological process that involves multiple molecules, it is likely that Bak may have dual roles in regulating cardiac remodeling. Our study cannot rule out other mechanisms in which Bak is involved; thus, future work is needed to explore the mechanism.

In conclusion, we demonstrate for the first time that Bak can effectively inhibit cardiac hypertrophy *in vivo*. More importantly, our study provides evidence for the application of Bak in the treatment of cardiac hypertrophy, which will contribute to the development of novel drugs for the treatment of cardiac hypertrophy.

## Abbreviations

β-MHC: β-myosin heavy chain
AB: aortic banding
ANP: atrial natriuretic peptide
Ang: angiotensin
AKT: protein kinase B
Bak: bakuchiol
BNP: B-type natriuretic peptide
CSA: cross-sectional area
Cdc: cyclin depentent kinase
FS: fractional shortening
GAPDH: glyceraldehyde-3-phosphate-dehydrogenase
HE: hematoxylin–eosin
HRT: hormone replacement therapy
IKKβ: inhibitor of κB kinase-β
IL-6: interleukin-6
IRI: ischemia reperfusion injury
MAPK: mitogen-activated protein kinase
MCP-1: monocyte chemoattractant protein 1
NFAT: nuclear factor of activated T cell
NF-KB: nuclear factor-kappa B
PSR: picrosirius red
PGC: peroxisome proliferator-activated receptor gamma coactivator
PDTC: pyrrolidinedithiocarbamic acid
PI3K: phosphatidylinositol 3-kinase
ROS: reactive oxygen species
TNF-α: tumor necrosis factor-α
